# Estimation of the Acoustic Transducer Beam Aperture by Using the Geometric Backscattering Model for Side-Scan Sonar Systems

**DOI:** 10.3390/s23042190

**Published:** 2023-02-15

**Authors:** Van Duc Nguyen, Ngoc Minh Luu, Quoc Khuong Nguyen, Tien-Dung Nguyen

**Affiliations:** School of Electrical and Electronic Engineering, Hanoi University of Science and Technology, Hanoi 100000, Vietnam

**Keywords:** side scanning sonar, underwater acoustic transducer, estimation of the beam aperture, underwater acoustic transducer

## Abstract

In this paper, we propose an algorithm for estimating the beam aperture of the acoustic transducers by using the geometric backscattering model for side-scan sonar systems. The geometric backscattering model is developed to describe the propagation paths of the signal transmitted from the transducers towards the seabed and backscatters to the hydrophones. To evaluate our proposed algorithm, we have developed a side-scan sonar system. The side-scan sonar system uses two transducers, operating on two different frequencies and focusing on two different wave beams, to scan the images of the seabed. The proposed algorithm provides the estimated beam apertures of each transducer. Our obtained results agree quite well with the parameters provided by the manufacturers. Moreover, these results are used to calibrate the scanned images. We provide the scanned sonar 3D images of the Dong Do lakebed, Vietnam, to justify our proposal.

## 1. Introduction

Object detection and imaging on the seafloor have gained great interest over time. Applications range from developing nautical charts, locating underwater hazards, or mapping the seafloor itself. However, technologies available for the purpose are very limited [1]. These days, one of the most feasible approaches is to use sonar (SOund and Navigation Ranging), which is a tool using sound waves to probe the underwater [2]. Herein, a towfish or vessel carries an array of transducers. These transducers emit acoustic signals into the water, bounce off the objects and return echoes to the array. The time from the emission to the reception of the corresponding echo is proportional to the distance it traveled. Then, both the distance to the object and the direction can be measured. Side-scan sonar system is a low-cost but effective tool to carry the mission [3].

The transducer typically emits a beam of signals into the water. Herein, the beam aperture plays an important role in developing underwater communication and side-scan sonar systems [4,5,6]. The accuracy of the beam aperture of the transducer determines the quality of the scanned sonar images, as well as the estimated size of the scanned areas [2]. Therefore, estimating such parameters is critical not only for commercial transducer manufacturers but also for research and development purposes where designing a transducer requires performing parameter verification.

In the literature, the beam aperture estimation is determined by the measurements of the beam pattern. For example, the study in [7] proposed a method for estimating the transmit beam pattern by calculating sound pressure [8]. The research work in [9] determines the beam pattern in the dependence of the transmit frequency ranging from 190 kHz to 400 kHz. The study in [10] provides a method to specify the beam pattern using the hydrophones mounted around the transmit transducer. Herein, the hydrophones are controlled by a servo system in such a way that the measured signal obtained by the hydrophones plots the beam pattern of the transmit transducer. The drawback of this method is the high complexity and high cost due to the complexity of the servo system. Moreover, it requires a huge effort to deploy.

This paper proposes an approach to estimate the beam aperture. Contrary to the research work in [10], our method does not require an extra servo system. Herein, we develop a geometric backscattering model for side-scan sonar signal transmission. Then, we propose an algorithm to process the received signal based on the proposed geometric backscattering model. The idea to develop our geometric backscattering model is based on the ray scattering theory [11,12,13]. Based on the analytical results of [14], we assume that the footprint of the side-scan sonar signal on the seabed has an elliptical form. This assumption is the background to developing our geometric backscattering model.

To evaluate our proposed method, we design and implement a side-scan sonar system, which operates at multiple frequencies. Afterward, we deploy the side-scan sonar system to scan the bottom surface of some lakes in Hanoi, Vietnam. In the system, we use commercial transducers with known aperture specifications and compare the estimated results with it to measure the error. Our geometric backscattering model is a mathematical model to calculate the beam aperture of transducers. Using this model, we develop an algorithm to estimate the transducer parameters from measurement data.

The rest of the paper is organized as follows: Section 2 provides an overview of the side-scan sonar architecture. To describe the traveling routes of the sonar signal, a novel geometric backscattering model is proposed in Section 3. Moreover, we propose an algorithm to estimate the beam aperture of the side-scan transducer in Section 4. The experimental results and discussion are provided in Section 5. Finally, Section 6 draws the conclusions.

## 2. Side-Scan Sonar System Overview

Sonar can be classified into passive and active technologies. While passive sonar does not emit any signal and is mainly used in military applications, active sonar sends an acoustic signal into the water and captures the echoes to compute the desired characteristics. The characteristics include depth, materials, texture types, biology, and geology features [15,16]. There are several types of active sonar systems: multibeam, side scan, split-beam, sub-bottom profiling, and synthetic aperture sonar. Among all, side scan sonar appears to be an economic and convenient tool and nowadays is widely applied in the surveys of rivers, lakes, and oceans [17].

**Figure 1:**
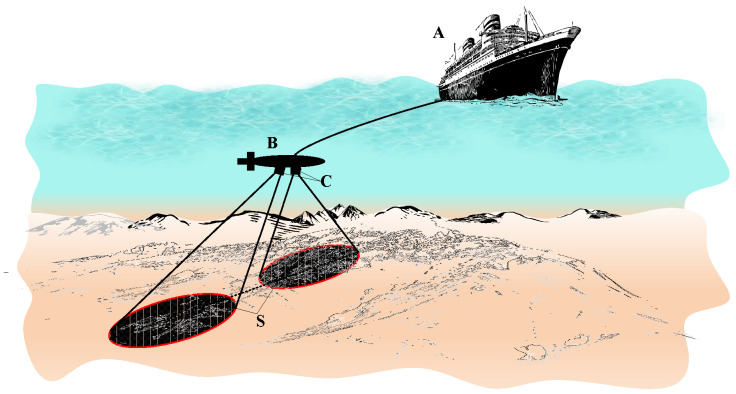
Side-scan sonar model deployed in our research. **A**: Towing vessel; **B**: Towfish; **C**: transducers; **S**: scan footprints on the seafloor.

To scan the seabed, the side-scan sonar technique used the information of the backscattered acoustic signals, which originally are emitted from two transducers mounted on either side of the towfish as depicted in Figure 1. The transducers can be also equipped on two opposite sides of the vessel’s hull, or underwater vehicles [18,19]. In this paper, we use the terms backscattered signal, echo signal, and reflected signal interchangeably.

Each transducer generates a beam of acoustic sound perpendicular to the main axis of the vessel or the towfish. When an acoustic signal is emitted and reached the seafloor, it will be backscattered. Among the backscattered signals, there will be several ones that arrive at the hydrophones. In our study, the first and the last measurable backscattered signals are used to estimate the beam aperture of the transmit transducer, which is a crucial parameter to reconstruct the scanned seafloor image.

The deployed side-scan sonar system in this research work is illustrated in Figure 1, where two acoustic transmitting transducers are mounted on two sides of the towfish, which is pulled by a towing vessel. Some notations of the side-scan sonar system in Figure 1 are explained as follows:

- **A**: Towing vessel.
- **B**: Towfish.
- **C**: Transducers mounted on two opposite sides of the towfish.
- **S**: Signal footprints of the conical sound beams formed by two transmit transducers.

Based on the side-scan sonar model depicted in Figure 1, we propose a backscattering geometry channel model and a signal processing algorithm to calculate the beam aperture of a transmit transducer. The input data for the algorithm is the measured data obtained from the backscattered signal from the receive transducer (acoustic sensor or hydrophone). The output of the proposed algorithm is the estimated beam aperture of the transmit transducer.

## 3. Proposed Geometric Backscattering Model for Calculating Transducer Beam Aperture

In this section, we introduce a geometric backscattering model for sound propagation in side-scan sonar systems. Based on this model, we can calculate the beam aperture of the transmit transducer from the back-scattered signals.

### 3.1. Description of Geometric Backscattering Model

**Figure 2:**
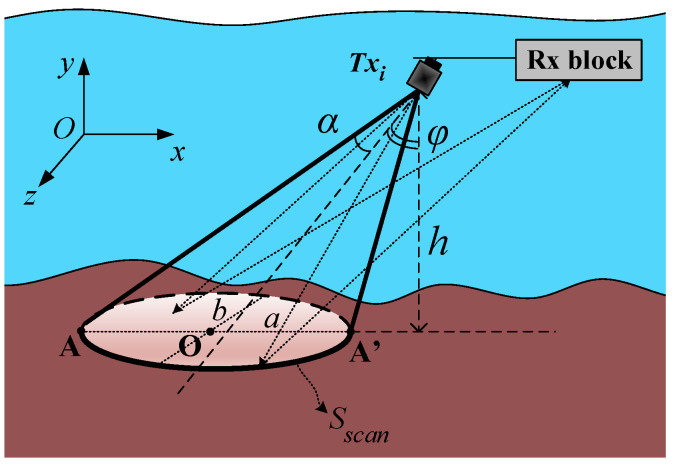
The geometric back-scattering model for signal propagation in side-scan systems.

The proposed geometric backscattering model in Figure 2 shows the model of one side of the side-scan sonar systems. The other side is symmetrical to this side, thus it is omitted to describe without loss of generality. The transducer designed for the side-scan sonar is a typical directional transducer with a conical beam shape. This type of transducer is used to develop our geometric backscattering model. The placement of the transmit transducer on the towfish is shown in Figure 1. The geometric backscattering for the acoustic signal propagation is modeled in Figure 2, where the towfish is assumed to move in the directionof Oz. The transmit and receive transducers are mounted on the plane Oxy, which is perpendicular to the moving direction Oz. As shown in Figure 2, the axis of the transmit transducer is deviated from the vertical axis by an angle φ. We denote *h* as the distance from the transducer to the seafloor and denote the opening angle of the transmit transducer as θ=2α. Then, the seabed surface obtained by the beam scanning should have an elliptical form. We denote *a* as the large diameter of this ellipse, and *b* as the small diameter. Thus, the scanned area is $S_{scan}=\pi ab$. The opening angle θ is called as the beam aperture of the transmit transducer.

According to the geometric backscattering channel model, the transducer will beam the sonar signal in an elliptical form. After reaching the sea floor, some of these signals will be backscattered to the receive transducer (hydrophone).

As depicted in Figure 2, the transmit and receive transducers are mounted on a line, which is parallel to the bottom plane. The distance between the transmit transducer and the receive one is $L_{i,j}$, where *i* and *j* are the transmit and the receive transducer indices, respectively.

### 3.2. Beam Aperture Calculation Method

**Figure 3:**
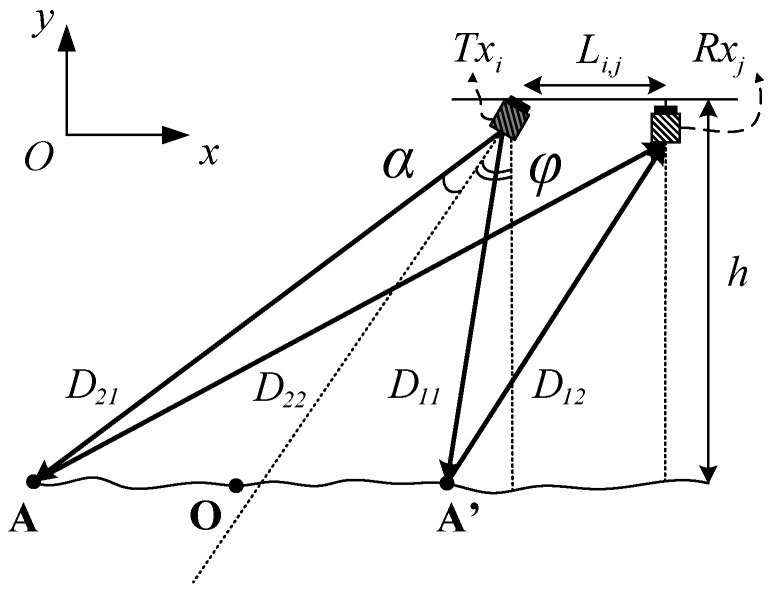
The mathematical geometric backscattering model observed in the vertical section.

The beam aperture of the transducer is calculated based on the mathematical model of the acoustic propagation, which is developed on the plane Oxy. As shown in Figure 3, the observed plane Oxy contains the transmit and receive transducer and is perpendicular to the bottom section of the elliptical beam. It is clear, that the longer diameter of the bottom section is parallel to the axis of the transmit and receive transducers. If we assume, that the observed sea floor is nearly flat (we can choose the area to do the experiment), then some sonar signals will be backscattered from the bottom section to the receivers. Based on the mathematical geometric model for the sound propagation in Figure 3, we can determine the length of the first seabed echo and the last seabed echo as $D_{1}=D_{11}+D_{12}$ and as $D_{2}=D_{21}+D_{22}$, respectively. The first seabed echo is the shortest path, which is reflected at the scattering object $A^{\prime }$ laying on the intersection between the plane Oxy and the bottom section. The last seabed echo is the longest path, which is reflected at the scattering object *A*.

In order to specify the beam aperture of the transmit transducer, θ=2α, we establish the equation with the unknown θ as follows:

(1) $$D=D_{2}-D_{1},$$

where *D* is the difference between the longest path and the shortest one. Based on the geometric backscattering model in Figure 3, it is straightforward to determine the downward path of the shortest echo route as follows:

(2) $$D_{11}=\frac{h}{\cos\left(\varphi -\alpha \right)}.$$

The backward path of this route is obtained by:

(3) $$D_{12}=\sqrt{{\left(L_{i,j}+\tan\left(\varphi -\alpha \right)h\right)}^{2}+h^{2}}$$

Similarly, we can derive the downward and the backward path of the longest echo route as:

(4) $$D_{21}=\frac{h}{\cos\left(\varphi +\alpha \right)},$$

and

(5) $$D_{22}=\sqrt{{\left(L_{i,j}+\tan\left(\varphi +\alpha \right)h\right)}^{2}+h^{2}}$$

In the above equations, *h* is the water depth, which can be estimated by using the sonar technique as discussed in Section 4. The Appendix A.2 provides a method to solve Equation (1) with unknown beam aperture θ.

## 4. Proposed Beam Aperture Estimation Algorithm

**Figure 4:**
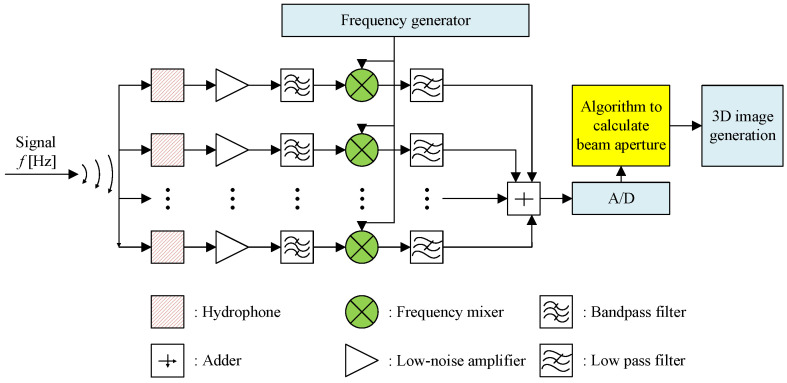
Block diagram of the receiver structure to estimate the beam aperture of the transmit transducer.

The block diagram of the receiver structure to estimate the beam aperture of the transducer is illustrated in Figure 4, where we use a number of parallel hydrophones to detect the sonar signal. The transmitted frequencies are $f_{c1}$ and $f_{c2}$. At the hydrophones, the received signals on hydrophone *j* will be mixed with a frequency $f_{mj}$ in order to distinguish signals received on that hydrophone. For example, by mixing frequency $f_{c1}$ and frequency $f_{mj}$, it will produce two frequency components, i.e., $|f_{c1}-f_{mj}|$, and $f_{c1}+f_{mj}$. The mixed signal will go through a low-pass filter to eliminate the high-frequency component. The low-frequency components on all the hydrophones are added together and fed into the audio port of the computer for processing. In the computer, a software program will separate the signals corresponding to each hydrophone (based on its frequency) and process them independently. Moreover, based on frequency, we can identify which signal belongs to which hydrophone.

**Figure 5:**
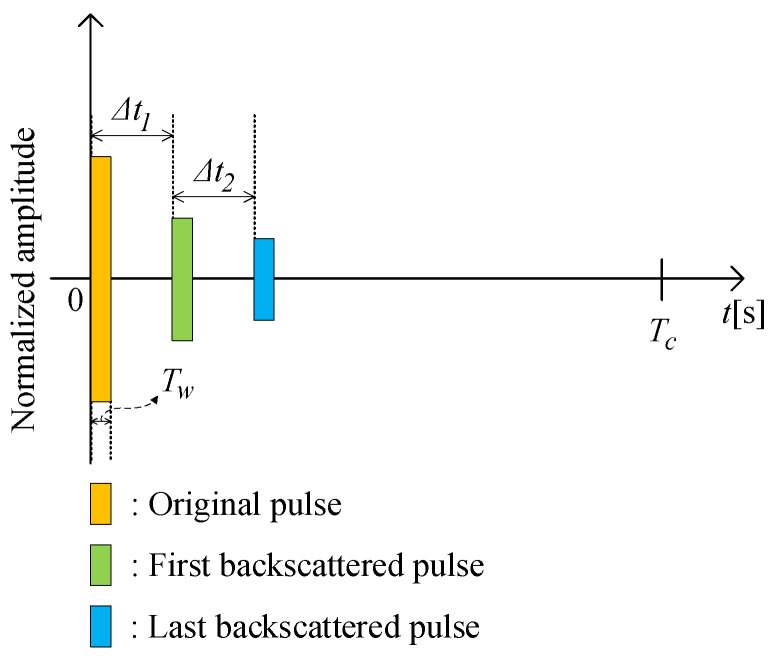
Received echo signal in one frame.

The system is composed of multiple transducers and multiple hydrophones. Indeed, this setup allows us to test the proposed beam aperture estimation algorithm on multiple transducers at the same time. The use of multiple hydrophones is to increase the chance of receiving the backscattered signals. On the other hand, it allows us to observe whether the beam aperture estimation depends on the distance between the transmitter and receiver or not. Putting aside the mentioned purposes, using one transducer and one hydrophone is enough. The distance between the transducer *i* and hydrophone *j* is $L_{i,j}$. This distance is fixed at deployment, which will be explained further in the experiment section, and the algorithm later will be based on this distance to calculate the beam aperture, as presented in Appendix A.2. The received signal after filtering the noise and synchronization is depicted in Figure 5, where $\Delta t_{1}$ is the first seabed echo received by the hydrophones. Thus, the water depth is attained by

(6) $$h=\frac{\Delta t_{1}\times c}{2}.$$

We denote $\Delta t_{2}$ as the time delay from the first seabed echo to the last one. The difference distance between the first seabed echo and the last one is derived by

(7) $$D=\Delta t_{2}\times c.$$

**Figure 6:**
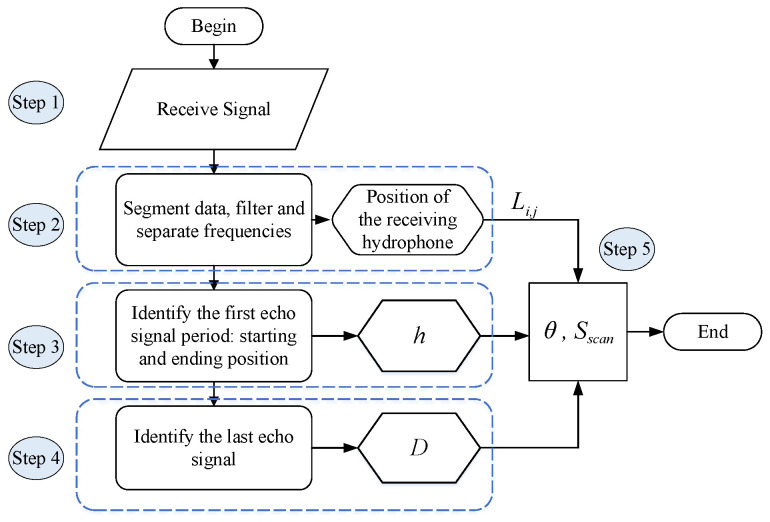
The proposed algorithm to estimate the beam aperture.

The proposed algorithm to estimate the beam aperture is shown in Figure 6, and is described asfollows:

***Step*** ***1***: Collect signals received from the hydrophones.
***Step*** ***2***: Synchronize the received signal following the period of the sonar signal $T_{c}$. One period of a sonar signal consists of a short pulse with the duration of $T_{w}$, and the zero padding signal. Besides transmitting the pulse towards the seabed, the pulse is also directly delivered to the receiving circuit as a reference signal (called the original pulse) to mark the starting point of the transmission period. At the receiving side, the original pulse is with maximal amplitude and the intervals between two pulses (i.e., $T_{c}$) are large enough so that the backscattered signals appear within the $T_{c}$ period, and do not overlap with signals in the next period. Based on the pulse amplitude and period, we can segment the received signals into individual frames (each frame contains received signals within a period $T_{c}$). After segmenting the received signal into each frame with a period of $T_{c}$, the signal on each hydrophone will be fed to frequency mixers to convert to a given bandwidth. The reason is that each hydrophone corresponds to a specific distance to the transducer (i.e., $L_{i,j}$), and knowing this distance is required to calculate the beam aperture. However, eventually, all the received signals from all hydrophones will be mixed and fed into the beam aperture calculation algorithm. Therefore, converting the received signal on a hydrophone to a distinct frequency band will allow the beam aperture calculation algorithm to later reversely identify the hydrophone. This is a technique to process the received signal on each hydrophone independently. Based on the signal bandwidth, we can identify which hydrophone the signals belong to. Then, the distance between the receive hydrophone and the transmit transducer can be obtained (i.e., $L_{i,j}$). This distance is required to compute the beam aperture later.
***Step*** ***3***: Identify the first seabed echo signal to calculate the water depth *h*. In each frame, the first echo signal is the signal at the desired frequency and has maximal amplitude following the emitted pulse in the time axis. The first echo signal can be detected by checking for the maximal signal amplitude, (excluding the original signal duration $T_{w}$ in Figure 5). Then, the algorithm computes the elapsed time since the original signal was transmitted towards the seabed $\Delta t_{1}$. The water depth *h* is derived from $\Delta t_{1}$ using Equation (6).

Then, the signal chunk consisting of the first echo signal will be removed from the frame: we already knew the beginning of the chunk, which is the maximal amplitude position; therefore, now we need to identify the ending position of the chunk. This chunk might consist of several other backscattered signals with similar propagation distance. Since the pulse traveled through a distance $D_{11}+D_{12}$ (approximately 2h), the attenuation will be proportional to the traveled distance. We derived a threshold Thr based on the water absorption and the traveling distance of the pulse as below.

(8) $$\mathrm{Thr}=10^{\frac{\alpha_{s}\times \left(D_{11}+D_{12}\right)\times 10^{-3}}{10}}\times A_{max}^{s},$$

where $\alpha_{s}$ is the absorption coefficient of sound in water, $A_{max}^{s}$ is the amplitude of the largest reflected signal. From the starting position of the first echo signal, the algorithm searches for the first signal position whose amplitude is smaller than the threshold. This is the ending position of the first echo signal. In Equation (8), the absorption coefficient of water is determined by [20,21,22]:

(9) $$\alpha_{s}=A_{1}P_{1}\frac{f_{1}f}{f_{1}^{2}+f^{2}}+A_{2}P_{2}\frac{f_{2}f}{f_{2}^{2}+f^{2}}+A_{3}P_{3}f^{2},$$

where the units of absorption coefficient $\alpha_{s}$, and the transmitting frequency *f* are [dB/km] and [kHz], respectively. $f_{1}$ and $f_{2}$ are the relaxation frequencies. The calculation of the factors in Equation (9) is described in detail in Appendix A.3.

***Step*** ***4***: After removing the first echo signal from the frame, now we can search for the last echo signal. Our empirical experiments reveal that in flat seabed conditions, one frame of received signals has the pattern described in Figure 5. A frame then has three parts: the original pulse, the first, and the last echo pulses. At this point, the original and the first echo pulses have been cut off from the frame. To identify the last echo signal, the algorithm detects the maximal amplitude position in the remaining frame. This allows us to determine $\Delta t_{2}$ since we already know the arrival time of the first echo signal (i.e., $\Delta t_{1}$). From here we can compute the distance difference between the first and last reflected paths *D* using Equation (7).
***Step*** ***5***: Based on the parameters $L_{i,j}$, *D*, and *h* obtained from ***Step 2*** to ***Step 4***, Equation (1) can be solved (see the solution in Appendix A.1) to calculate the beam aperture $\theta^{\circ }$ and the bottom scanned area $S_{scan}$, observed by each hydrophone.

Supposing that the received signal consists of *K* frames, the estimation performance of the beam aperture of each transducer measured at each hydrophone is increased by averaging all the estimated results in each frame as follows [23]:

(10) $$\langle \theta \rangle =\frac{1}{K}\sum_{k=1}^{K}\theta_{k}=\frac{1}{K}\left(\theta_{1}+\theta_{2}+\ldots +\theta_{K-1}+\theta_{K}\right),$$

where 〈θ〉 is the averaged beam aperture estimated at each hydrophone, *K* is the number of frames to be split for processing, $\theta_{k}=2\alpha_{k}$ is the calculated beam aperture at the $k^{\mathrm{th}}$ frame of the hydrophone. The variance of the estimated beam aperture is:

(11) $$\sigma_{\theta }=\sqrt{\frac{1}{K-1}\times \sum_{k=1}^{K}{\left(\theta_{k}-\langle \theta \rangle \right)}^{2}}$$

The relative estimation error of the beam aperture is defined by:

(12) $$E=\frac{|\langle \theta \rangle -\theta_{fac}|}{\theta_{fac}},$$

where $\theta_{fac}$ is the beam aperture provided by the manufacturer.

Note that, *h* and *D* are calculated at different times. Herein, assuming that the transducer emits a signal at time 0, then *h* and $D_{1}$ are calculated at time $\Delta t_{1}$, and $D_{2}$ is calculated at time $\Delta t_{1}+\Delta t_{2}$. Theoretically, during the times, the position of the boat might be shifted, resulting in the changes in *h*, and therefore, affecting the estimation of θ. However, we will show in the experimental results section that such change is negligible and should not harm the beam aperture estimation result.

## 5. Experimental Results and Discussions

### 5.1. Testbed Scenario

**Figure 7:**
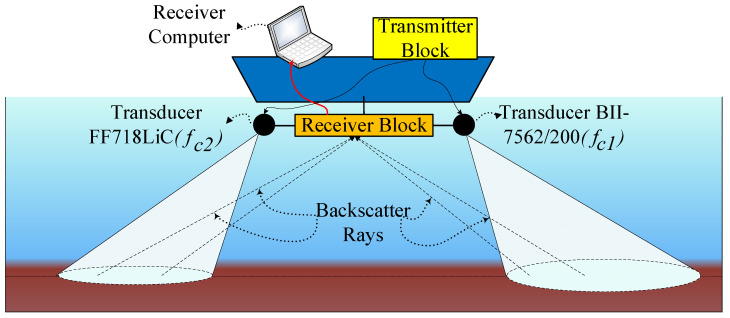
Testbed block structure.

**Figure 8:**
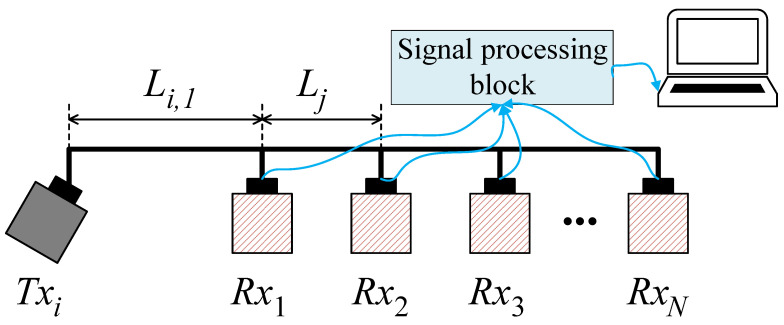
Scheme for mounting the transmit transducer and hydrophones in one side of the boat.

The testbed block structure is depicted in Figure 7, where two transmit transducers are mounted on two opposite sides of a small boat. Eight hydrophones are mounted in line with the two transmit transducers. The transmit transducers deviate their directions to outside the boat’s areas by an angle φ compared to the vertical direction. The setup is illustrated in Figure 8. Each hydrophone will sense the echo signals to compute the transducer beam aperture, and scan the seabed.

The main goal is to measure the beam aperture of the transducers. The beam aperture can be estimated by emitting one pulse, and we conducted the experiment in shallow water. As a consequence, the traveling time of the emitted signal is short, during which we can assume that the boat does not move. The shallow water (approximately 10 m) also allowed us to assume that the other conditions (e.g., water temperature, salinity, waves) are of minimal effect on the measurement.

**Figure 9:**
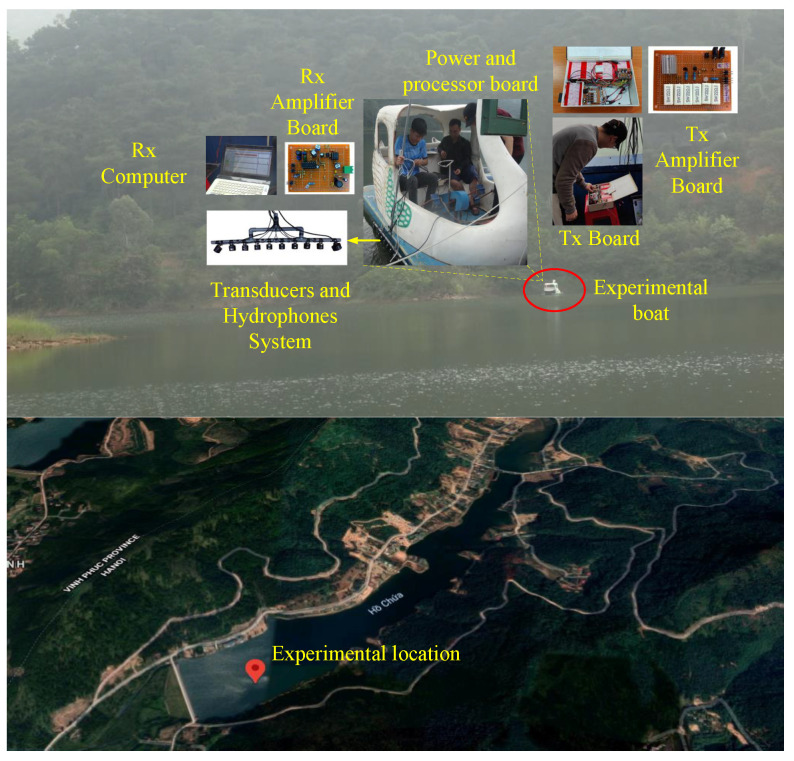
Experiments conducted on 16 October 2021 in Dong Do lake, Hanoi, Vietnam.

**Figure 10:**
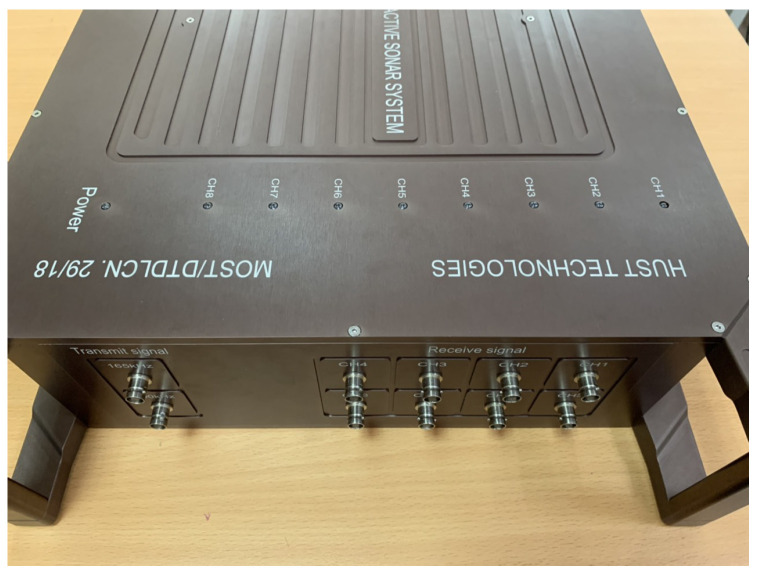
Our implemented side-scan sonar.

Our real testbed to estimate the beam aperture of the transducer, and to scan the sonar 3D image is shown in Figure 9, and our implemented hardware side scan sonar equipment is given in Figure 10.

### 5.2. System Parameters and Environmental Conditions

The experiments were conducted on 16 October 2021 in Dong Do lake, Hanoi, Vietnam. This lake is fresh and shallow. The lake bottom is relatively flat. Table 1 describes the system parameters, while Table 2 provides the experimental conditions. All the received signals are sampled at 192 kHz, i.e., for a period 0.1 s, there will be 19,200 samples. The distance between any two modules (transducers, hydrophones) is 0.09 m. The emitting angle of the transducer is φ=30 degree. Note that, for estimating the beam aperture of the transducer, we need to know the depth *h*, the difference $D=D_{2}-D_{1}$, and the emitting angle φ. In an ideal situation, *h* should be a constant, i.e., can be achieved if the boat does not move. In fact, due to the wind or water flow, the boat could be shifted but this shift does not cause a notable effect on the result. We will discuss this further in the following subsection.

One transmit transducer is the BII-7562/200 [24], transmitting the sonar signal at the frequency $f_{c1}=165$ kHz. The other one is the transducer FF718LiC [25], transmitting the sonar signal at the frequency $f_{c2}=200$ kHz. The salinity and pH of Dong Do Lake are adopted from the research published in [26]. Since the two transducers emit two different frequencies, the water absorption for these signals is different. As a consequence, the threshold Thr should be different for the backscattered signals of each transducer. The backscattered signals are separated based on their frequencies. Therefore, the system can check the received signal amplitude against Thr on each frequency channel.

At the receiving system, signals received at each hydrophone will be mixed with a different frequency from 120 kHz to 150 kHz, one per hydrophone. Since the emitting signals are at 165 kHz and 200 kHz, after the low pass filter, the resulting signals will be in the frequencies from 15 kHz to 80 kHz. For example, hydrophone $R_{x1}$ receives backscattered signals at 165 kHz and 200 kHz. Mixing the signals with the frequency 120 kHz will produce four frequencies: 320 kHz, 285 kHz, 80 kHz, and 45 kHz. The low pass filter will retain the 80 kHz and 45 kHz only. Similarly, if signals received at the last hydrophone $R_{x8}$ are mixed with the frequency 150 kHz, then the filter signals will be at 15 kHz and 50 kHz. The mixed frequencies on the hydrophones are evenly-spaced. In order to reduce the number of generated frequencies, one can divide the receiving hydrophones into two groups, and each group can be mixed and directed to a channel in the audio input port of the computer (an audio port typically has two input channels).

### 5.3. Measurement Data Analysis Results

#### 5.3.1. Backscatterred Signal Echoed Time

**Figure 11:**
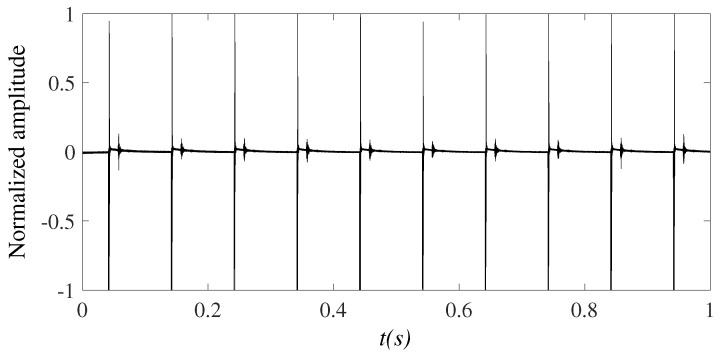
Received seabed echo signals.

**Figure 12:**
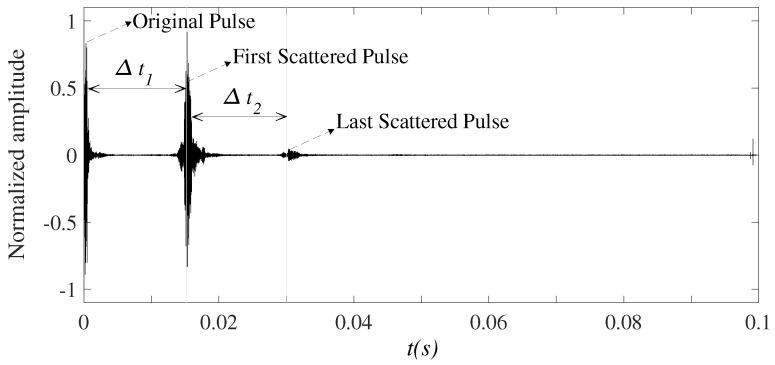
A frame of received seabed echo signals.

The transducer continuously emits one pulse after $T_{c}$ (seconds). There are multiple echoed pulses, for every emitted pulse, arriving at the hydrophone arrays at different delays and amplitudes. We refer to a *frame* as a set of signals received at the hydrophones for one emitted pulse, this includes the original signal (directly transmitted to the receiving system) and the backscattered signals. Figure 11 shows a window of received signals which contains 10 frames, corresponding to 10 times the transducer transmits a pulse into the water. The period $T_{c}$ is sufficiently big so that the frames are distinguishable. The received signal is passed through a bandpass filter in order to remove the out-of-band noise if any. Afterwards, the received signal is segmented into individual frames, each consisting of the original pulse, and the first and the last backscattered pulses as plotted in Figure 12. In this experiment, the time from the original pulse to the last backscattered pulse is approximately equal to 0.03 s. The sidelobes of the transducers could be received at the hydrophones as well. However, since the transmitted pulse is short (0.4 ms) and the propagation speed of the acoustic wave in the water is low (around 1500 m/s), if the water depth is 10 m, then the minimum propagation time is around 13 ms. In other words, the first backscattered signal to arrive at the receiving system is not earlier than 13 ms. This time is large enough to make the backscattered signals distinguishable from the sidelobes, which should appear close to the original pulse.

Based on the original pulse and the first backscattered pulse, we can obtain the water depth *h*. Herein, the interval between the transmission of the transducer and the last echo signals is about 0.03 s. During this period, we can assume that the boat stays still on the surface. For example, if the boat floats at 0.5 m/s, then after 0.03 s, its shifted distance is 0.015 m, which is negligible.

The large and small diameters of the bottom elliptical section are calculated by Equation (A1), and Equation (A4), respectively. The variance of the estimated beam aperture is obtained by using Equation (11). The estimated results for two transducers and the corresponding scanned areas are summarized in Table 3.

#### 5.3.2. Measurement Error

Based on the first backscattered pulse and the last backscattered one, we can calculate the distance difference of these paths, i.e., *D*. Applying the proposed algorithm by using the obtained *h* and *D*, together with the parameters given in Table 2, we can calculate the transducer beam aperture and the size of the scanned area.

**Figure 13:**
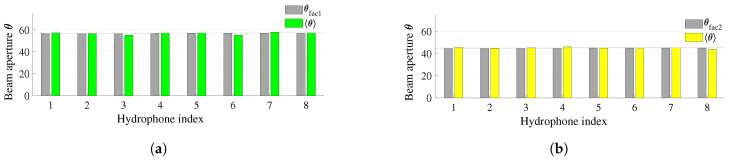
A comparison of the estimated beam aperture with the parameters provided by the manufacturer: (**a**) Transducer type 1 (BII-7562/200); (**b**) Transducer type 2 (FF718LiC).

**Figure 14:**
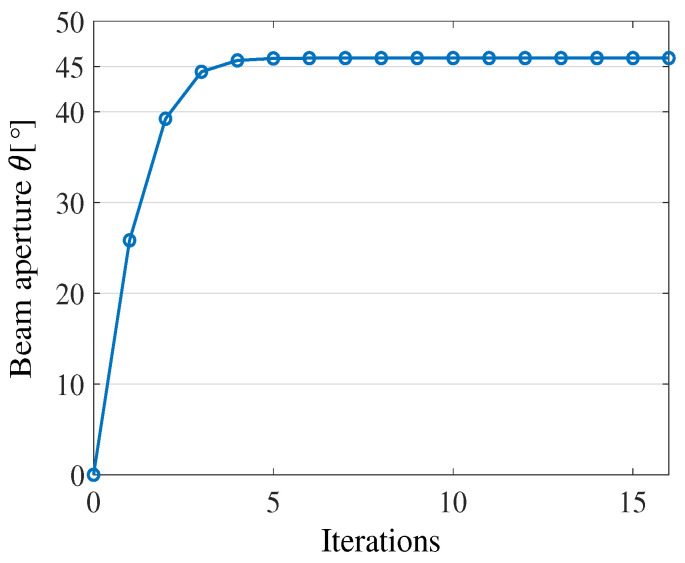
Beam aperture estimation of FF718LiC transducer.

Figure 13 compares the beam apertures of two transducers estimated on eight hydrophones with those ones given by the manufacturer. The first transducer is BII-7562 (Benthowave manufacture) operated at the frequency of 165 KHz, whereas the latter one is the FF718LiC (Lucky star manufacture) worked at the frequency of 200 KHz. The corresponding beam apertures of the two transducers are $57^{\circ }$ and $45^{\circ }$, respectively. On the x-axis is the index of each hydrophone. Since the two transmit transducers are mounted at opposite ends of the hydrophone array (Figure 7), the distances from each hydrophone to the transmit transducer in Figure 13a,b are different. For example, in Figure 13a the distance from hydrophone 1 to the transmit transducer (type 1) is 0.09 m, whereas the distance from hydrophone 1 to the transmit transducer (type 2) in Figure 13b is 0.72 m. Typically, $L_{1,j}=0.81-L_{2,j}$. It can be seen that the obtained results are very close to the manufacturer’s parameters. Assume that we have h=10.05, ϕ=30, $L_{2,7}=0.63$, and D=13.53. Putting the numbers in Equation (A9) in Appendix A.2, and running the Newton–Raphson method we will see the value of α converges after four iterations as in Figure 14.

**Figure 15:**
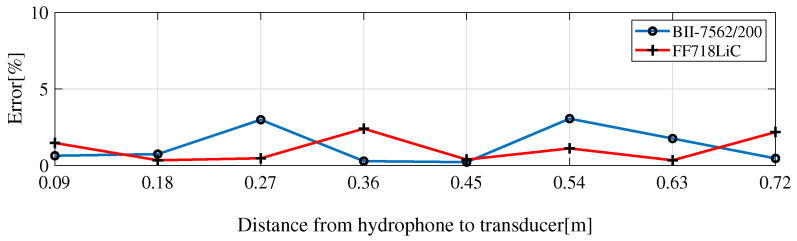
Beam aperture estimation error.

Figure 15 presents the measurement error in percentage. On the x-axis is the distance from the receive hydrophone to the transmit transducer. The peak errors are smaller than 3%. Most of the time, the errors do not cross over 1%.

#### 5.3.3. Use Case: 3D Lakebed Image Reconstruction

As mentioned before, beam aperture estimation plays an important role in underwater surveys. In our system, we made a transducer and the more accurate the estimation of the beam aperture is, the more accurate we can reconstruct the 3D image of the lakebed.

**Figure 16:**
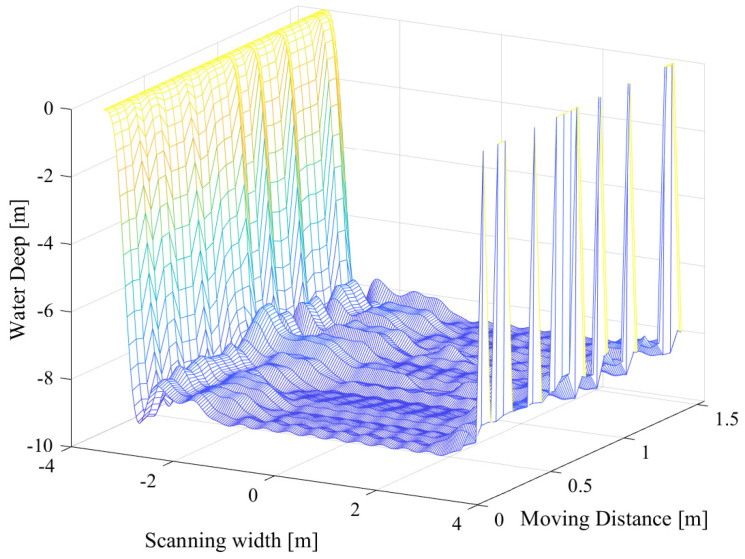
3D scanned image of the Dong Do lakebed.

Figure 16 shows a 3D sonar picture of the Dong Do lakebed obtained by our side-scan system. The estimated beam aperture of the transducer is adopted in the implemented system to calibrate this image. In this experiment, the boat constantly moves in a straight line and the measured depth is around 10 m. The scanned bottom width is about 3 m each side.

## 6. Conclusions

This paper proposed a geometric backscattering model for describing the traveling routes of the seabed echo signals, an algorithm to estimate the beam aperture of the transmit transducer. We have deployed a testbed to acquire data, and scan the Dong Do lakebed. The estimated transducer beam aperture obtained by our method closely approximates the specification provided by the manufacturers. The obtained transducer parameters are crucial factors to calibrate the 3D sonar images. As a result, our platform effectively measures the depth and renders the 3D bottom surface of the Dong Do lake.

## Figures and Tables

**Table 1:** System parameters.

Parameter	Value
Pulse period $T_{c}$	0.1 s
Pulse width $T_{w}$	0.4 ms
Sampling frequency	192 kHz
Recorded data duration	10 s
Number of transducers *M*	2
Number of hydrophones *N*	8
Distance between transducer and the first hydrophone $L_{i,1}$	0.09 m
Distance between two adjacent hydrophones $L_{j}$	0.09 m
Transducer elevation angle relative to vertical φ	$30^{\circ }$
$Tx_{1}$ carrier frequency $f_{c1}$	165 kHz
$Tx_{2}$ carrier frequency $f_{c2}$	200 kHz
Mixed frequencies	120 kHz–150 kHz

**Table 2:** Environmental conditions.

Weather	Sunshine
Water temperature	25 °C
pH indicator	7
Salinity S	0.5

**Table 3:** Estimated transducer parameters and scanned areas calculation results.

Transducer Type	Operating Frequency	〈θ〉	$\sigma_{\theta }^{\circ }$	a[m]	b[m]	$S_{\mathrm{scan}}\left[m^{2}\right]$
BII-7562	165 kHz	56.76	0.98	8.03	6.60	166.5
FF718LiC	200 kHz	45.03	0.42	5.89	4.96	91.92

**Figure A1:**
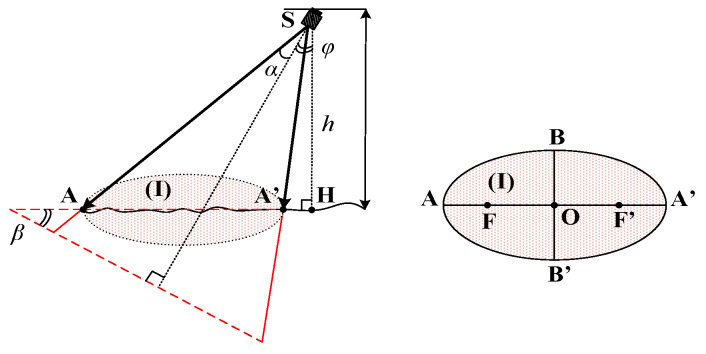
Calculation diagram of elliptical bottom sweep.

## Data Availability

Not applicable.

## Appendix A

### Appendix A.1. Calculation of the Area of Scanned Seabed

According to [27], the area of the scanned seabed can be calculated based on the area of the elliptical bottom section, which is created by the conical beam of the transducer with the seabed. To calculate the area of the elliptical bottom section, i.e., $S_{scan}=\pi ab$, we have to calculate the large radius *a* and the small radius *b*.

Based on Figure A1, the large radius *a* is OA, and the small radius *b* is OB. It is clear, that the large radius can be calculated as:

(A1) $$OA=\frac{AH-A^{\prime }H}{2},$$

where

(A2) AH=htan(φ+α),

and

(A3) $$A^{\prime }H=h\tan\left(\varphi -\alpha \right).$$

The small radius of the elliptical bottom section can be obtained by:

(A4) $$OB=\sqrt{OA^{2}-OF^{2}},$$

where OF is the focal length of the ellipse, and, according to [27], is calculated by

(A5) $$OF=\frac{OA}{\cos\left(\alpha \right)}\sin\left(\beta \right).$$

The angle β is equal to ϕ.

(A6) $$\beta =90^{\circ }-\alpha -\hat{SAH}.$$

(A7) $$\hat{SAH}=\arctan\left(h/AH\right).$$

### Appendix A.2. Solving the Equation with the Unknown Beam Aperture by Using the Newton–Raphson Method

We apply the Newton–Raphson method in [28] to solve Equation (1) with the unknown beam aperture α as follows:

(A8) $$\begin{array}{cc} f(\alpha )= & \frac{h}{\cos\left(\varphi +\alpha \right)}-\frac{h}{\cos\left(\varphi -\alpha \right)}\\ \; & +\sqrt{{\left(L_{i,j}+\tan\left(\varphi +\alpha \right)h\right)}^{2}+h^{2}}\\ \; & -\sqrt{{\left(L_{i,j}+\tan\left(\varphi -\alpha \right)h\right)}^{2}+h^{2}}-D=0. \end{array}$$

The derivative of the function f(α) with respect to the variable α gives

(A9) $$\begin{array}{cc} f^{\prime }\left(\alpha \right)= & \frac{h\sin(\varphi +\alpha )}{\cos^{2}\left(\varphi +\alpha \right)}+\frac{h\sin(\varphi -\alpha )}{\cos^{2}\left(\varphi -\alpha \right)}\\ \; & +\frac{h[\left(L_{i,j}+\tan\left(\varphi +\alpha \right)h\right)]}{\cos^{2}\left(\varphi +\alpha \right)\sqrt{{\left(L_{i,j}+\tan\left(\varphi +\alpha \right)h\right)}^{2}+h^{2}}}\\ \; & +\frac{h[\left(L_{i,j}+\tan\left(\varphi -\alpha \right)h\right)]}{\cos^{2}\left(\varphi -\alpha \right)\sqrt{{\left(L_{i,j}+\tan\left(\varphi -\alpha \right)h\right)}^{2}+h^{2}}}. \end{array}$$

Supposed that $\alpha_{0}$ is the initial solution for the equation given above, then the next approximation to close to the true solution will be:

(A10) $$\alpha_{1}=\alpha_{0}-\frac{f(\alpha_{0})}{f^{\prime }\left(\alpha_{0}\right)}.$$

This approximation will be continued until we obtain the true value of the solution [29], i.e.,

(A11) $$\alpha_{n+1}=\alpha_{n}-\frac{f(\alpha_{n})}{f^{\prime }\left(\alpha_{n}\right)}.$$

### Appendix A.3. Calculation of the Water Absorption Coefficients

Assuming that the signal propagated in water is only affected by absorption, then after a distance, $M_{d}$ will suffer attenuation due to absorption in water, and the signal will attenuate in amplitude with a magnitude satisfying inequality:

(A12) $$\frac{A_{M_{d}}^{s}}{A_{max}^{s}}\leq R_{absortion_{M_{d}}}$$

where $A_{max}^{s}$ is the amplitude of the original signal, $A_{M_{d}}^{s}$ is the reflected signal amplitude when the signal transmit in water with a distance $M_{d}$ coming later from $A_{max}^{s}$, $R_{absortion_{M_{d}}}$ is the attenuation ratio of the $A_{M_{d}}^{s}$ signal compared to $A_{max}^{s}$. This ratio is determined based on the formula for attenuation coefficient due to absorption in water $\alpha_{s}$. The absorption coefficient in water is determined by a model [22] as follows:

(A13) $$\alpha =C_{1}\frac{f_{1}f}{f_{1}^{2}+f^{2}}+C_{2}\frac{f_{2}f}{f_{2}^{2}+f^{2}}+C_{3}f^{2}$$

The contributions from the two relaxation processes are represented in the first two components of this formula, while the pure water viscosity is represented in the third term. Experiments in a lab or at sea are used to estimate the relaxation frequencies $f_{i}$ and coefficients $C_{i}$, which are dependent on temperature, hydrostatic pressure, and salinity.

The old model used to determine this coefficient is determined by [30,31,32]; however, it is only determined for low carrier frequencies below 50 kHz:

$$\alpha =\left[\frac{0.11}{1+f^{2}}+\frac{44}{4100+f^{2}}\right]f^{2}$$

So in the case of the system of this paper, we use the more popular Francois–Garrison model [20,21] for the carrier frequency higher than 50 kHz. Hence, the absorption coefficient is divided into three parts to reflect the contributions of boric acid, magnesium sulfate, and pure water, respectively:

(A14) $$\alpha_{s}=A_{1}P_{1}\frac{f_{1}f}{f_{1}^{2}+f^{2}}+A_{2}P_{2}\frac{f_{2}f}{f_{2}^{2}+f^{2}}+A_{3}P_{3}f^{2},$$

where $\alpha_{s}$ [dB/km] is the attenuation, *h* is the depth, in m; pH is the concentration of hydrogen ions in a water-based solution; *S* is the salinity, in per thousand; *T* is the temperature, in °C; and *f* is carrier frequency, in kHz.

The contribution of boric acid $B{\left(OH\right)}_{3}$ in the first term of the Equation (A14) reads:

(A15) $$\left\{\begin{array}{c} A_{1}=\frac{8.86}{c_{\alpha_{s}}}10^{\left(0.78pH-5\right)}\\ P_{1}=1\\ f_{1}=2.8\sqrt{\frac{S}{35}}10^{\left(4-1245/(T+273)\right)}\\ c_{\alpha_{s}}=1412+3.21T+1.19S+0.0167h \end{array}\right.$$

The contribution of magnesium sulfate $MgSO_{4}$ in the second term of the Equation (A14) reads:

(A16) $$\left\{\begin{array}{c} A_{2}=21.44\frac{S}{c_{\alpha_{s}}}\left(1+0.025T\right)\\ P_{2}=1-1.37\times 10^{-4}h+6.2\times 10^{-9}h^{2}\\ f_{2}=\frac{8.17\times 10^{\left(8-1990/(T+273)\right)}}{1+0.0018(S-35)} \end{array}\right.$$

The contribution of pure water viscosity in the last term of the Equation (A14) reads:

(A17) $$\left\{\begin{array}{c} P_{3}=1-3.83\times 10^{-5}h+4.9\times 10^{-10}h^{2}\\ T<20{\;}^{\circ }C\Rightarrow A_{3}=4.937\times 10^{-4}-2.59\times 10^{-5}T\\ +9.11\times 10^{-7}T^{2}-1.5\times 10^{-8}T^{3}\\ T>20{\;}^{\circ }C\Rightarrow A_{3}=3.964\times 10^{-4}-1.146\times 10^{-5}T\\ +1.45\times 10^{-7}T^{2}-6.5\times 10^{-8}T^{3} \end{array}\right.$$

From the parameters in the experimental environment, the set of operators used for Equation (A14) is as follows:

(A18) $$\left\{\begin{array}{c} A_{1}=\frac{8.86}{c_{\alpha_{s}}}10^{\left(0.78pH-5\right)}\\ P_{1}=1\\ f_{1}=2.8\sqrt{\frac{S}{35}}10^{\left(4-1245/(T+273)\right)}\\ c_{\alpha_{s}}=1412+3.21T+1.19S+0.0167h\\ A_{2}=21.44\frac{S}{c_{\alpha_{s}}}\left(1+0.025T\right)\\ P_{2}=1-1.37\times 10^{-4}h+6.2\times 10^{-9}h^{2}\\ f_{2}=\frac{8.17\times 10^{\left(8-1990/(T+273)\right)}}{1+0.0018(S-35)}\\ P_{3}=1-3.83\times 10^{-5}h+4.9\times 10^{-10}h^{2}\\ A_{3}=3.964\times 10^{-4}-1.146\times 10^{-5}T+1.45\times 10^{-7}T^{2}\\ -6.5\times 10^{-8}T^{3} \end{array}\right.$$

Based on the formula for converting logarithmic (dB) to linear and $\alpha_{s}$ [dB/km] and $M_{d}$ [m] we have:

(A19) $$R_{absortion_{M_{d}}}=10^{\frac{\alpha_{s}\times \left(M_{d}\times 10^{-3}\right)}{10}}$$

From Equations (A12) and (A19):

(A20) $$\frac{A_{M_{d}}^{s}}{A_{max}^{s}}\leq 10^{\frac{\alpha_{s}\times \left(M_{d}\times 10^{-3}\right)}{10}}\Rightarrow A_{M_{d}}^{s}\leq 10^{\frac{\alpha_{s}\times \left(M_{d}\times 10^{-3}\right)}{10}}\times A_{max}^{s}$$

In this paper, with $M_{d}=D_{1}=D_{11}+D_{12}$ is the propagation distance of first scattering pulse, denote $\mathrm{Thr}=10^{\frac{\alpha_{s}\times \left(M_{d}\times 10^{-3}\right)}{10}}\times A_{max}^{s}$ so the threshold level equation is proved.
